# CXCL16 Deficiency Attenuates Renal Injury and Fibrosis in Salt-Sensitive Hypertension

**DOI:** 10.1038/srep28715

**Published:** 2016-06-29

**Authors:** Hua Liang, Zhiheng Ma, Hui Peng, Liqun He, Zhaoyong Hu, Yanlin Wang

**Affiliations:** 1 Selzman Institute for Kidney Health and Section of Nephrology, Department of Medicine, Baylor College of Medicine, Houston, Texas, USA; 2 Department of Anesthesiology, Affiliated Foshan Hospital of Sun Yat-sen University, Foshan, China; 3 Section of Nephrology, Department of Medicine, Shuguang Hospital, Shanghai, China; 4 Section of Nephrology, Department of Internal Medicine, the Third Affiliated Hospital of Sun Yat-sen University, Guangzhou, China; 5 Center for Translational Research on Inflammatory Diseases (CTRID) and Renal Section, Michael E. DeBakey VA Medical Center, Houston, Texas, USA

## Abstract

Inflammation plays an important role in the pathogenesis of hypertensive kidney disease. However, the molecular mechanisms underlying the induction of inflammation are not completely understood. We have found that CXCL16 is induced in the kidney in deoxycorticosterone acetate (DOCA)-salt hypertension. Here we examined whether CXCL16 is involved in DOCA-salt-induced renal inflammation and fibrosis. Wild-type and CXCL16 knockout mice were subjected to uninephrectomy and DOCA-salt treatment for 3 weeks. There was no difference in blood pressure at baseline between wild-type and CXCL16 knockout mice. DOCA-salt treatment resulted in significant elevation in blood pressure that was comparable between wild-type and CXCL16 knockout mice. CXCL16 knockout mice exhibited less severe renal dysfunction, proteinuria, and fibrosis after DOCA-salt treatment compared with wild-type mice. CXCL16 deficiency attenuated extracellular matrix protein production and suppressed bone marrow–derived fibroblast accumulation and myofibroblast formation in the kidneys following DOCA-salt treatment. Furthermore, CXCL16 deficiency reduced macrophage and T cell infiltration into the kidneys in response to DOCA-salt hypertension. Taken together, our results indicate that CXCL16 plays a key role in the pathogenesis of renal injury and fibrosis in salt-sensitive hypertension through regulation of bone marrow–derived fibroblast accumulation and macrophage and T cell infiltration.

Hypertension is a common cause of chronic kidney disease (CKD)^1^. Hypertensive kidney disease is characterized by glomerular and interstitial fibrosis, tubular atrophy, and inflammation^2^. Renal interstitial fibrosis is characterized by accumulation of activated fibroblasts and overproduction of extracellular matrix proteins (ECM), which result in progressive loss of kidney function to end-stage renal disease^3^,^4^. Currently, therapeutic options for this devastating disorder are scarce and often ineffective except for life-long dialysis or kidney transplantation^4^. Thus, an improved understanding of the molecular mechanisms involved in the initiation and progression of CKD may lead to development of rational strategies to treat this disorder and prevent its progression.

Salt intake is one of the main environmental factors contributing to the development of hypertension^5^. Salt-sensitive hypertension can be induced by deoxycorticosterone acetate (DOCA) and salt. Salt-sensitive hypertension accounts for about 30% of primary hypertension in clinical settings^6^,^7^. Salt-sensitive hypertension has been shown to be a major cause of renal injury and have a greater incidence of end-stage renal disease^5^,^8^. However, the underlying mechanisms underlying the pathogenesis of salt-sensitive hypertensive kidney disease are incompletely understood.

Recent studies have shown that circulating cells plays an important role in the pathogenesis of hypertensive end organ damage including the kidney^9^,^10^,^11^,^12^. The infiltration of circulating cells into sites of injury is mediated by locally produced chemokines through interaction with their respective receptors^13^. Chemokine (C-X-C motif) ligand 16 (CXCL16) is a recently discovered cytokine belonging to the CXC chemokine subfamily^14^. CXCL16 exists not only in a transmembrane form as an adhesion molecule, but also in a soluble CXCL16 form that mediates infiltration of circulating cells into sites of injury^15^,^16^. However, the role of CXCL16 in DOCA-salt-induced hypertensive kidney disease is not known. We have found that CXCL16 is upregulated in the kidney in response to DCOA-salt hypertension. Therefore, we investigated the role of CXCL16 in the pathogenesis of DOCA-salt hypertensive renal injury and fibrosis using CXCL16 knockout (KO) mice. Our results have shown that genetic disruption of CXCL16 protects the kidney from DCOA-salt hypertensive renal injury and fibrosis through inhibiting bone marrow–derived fibroblast accumulation and macrophage and T cell infiltration.

## Results

### CXCL16 is Induced in the Kidney in DOCA-salt Hypertension

**Figure 1:**
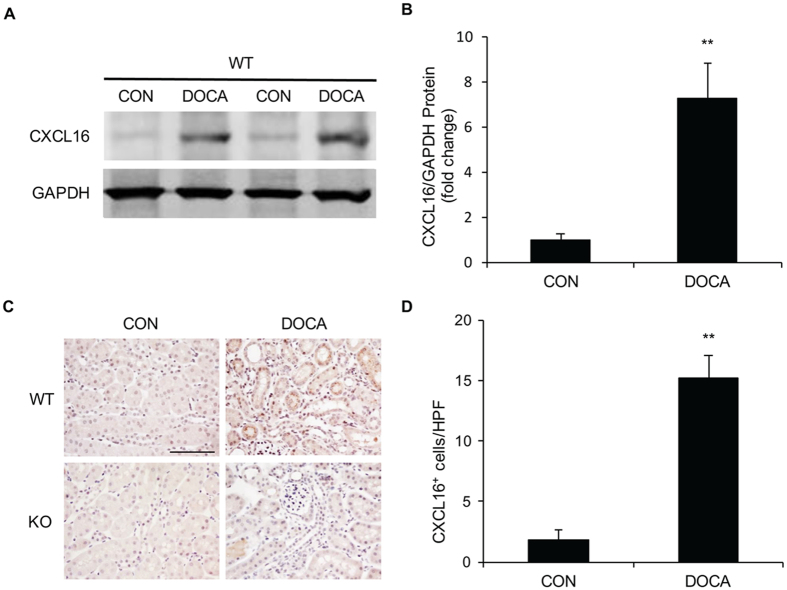
CXCL16 is upregulated in the kidney following DOCA-salt treatment. (**A**) Representative western blot shows CXCL16 protein levels in the kidneys of WT after vehicle or DOCA-salt treatment. (**B**) Quantitative analysis of CXCL16 protein expression in the kidneys. **P < 0.01 vs. WT CON. n = 6 in each group. (**C**) Representative photomicrographs of kidney sections stained for CXCL16 (brown) and counterstained with hematoxylin (blue). Scale bar, 50 μm. (**D)** Quantitative analysis of CXCL16-positive cells in the kidney. **P < 0.01 vs. WT CON. n = 6 in each group. HPF, high-power field.

We first determined whether CXCL16 is induced in the kidney in a mouse model of DOCA-salt hypertension. Western blot analysis revealed that the protein levels of CXCL16 were upregulated significantly in the kidneys after 3 weeks of DOCA-salt treatment compared with controls (Fig. 1A,B). Immunohistochemical staining was performed to examine the cell type responsible for the induction of CXCL16. The results showed that CXCL16 protein was expressed at a low level in tubular epithelial cells of control kidney, which was markedly induced in tubular epithelial cells of WT mice received DOCA-salt. Of note, no positive staining for CXCL16 was detected in the kidney of CXCL16 KO mice, confirming genetic disruption of the CXCL16 gene (Fig. 1C,D). These data indicate that DOCA-salt hypertension induces CXCL16 expression in the kidney.

### Blood Pressure

**Figure 2:**
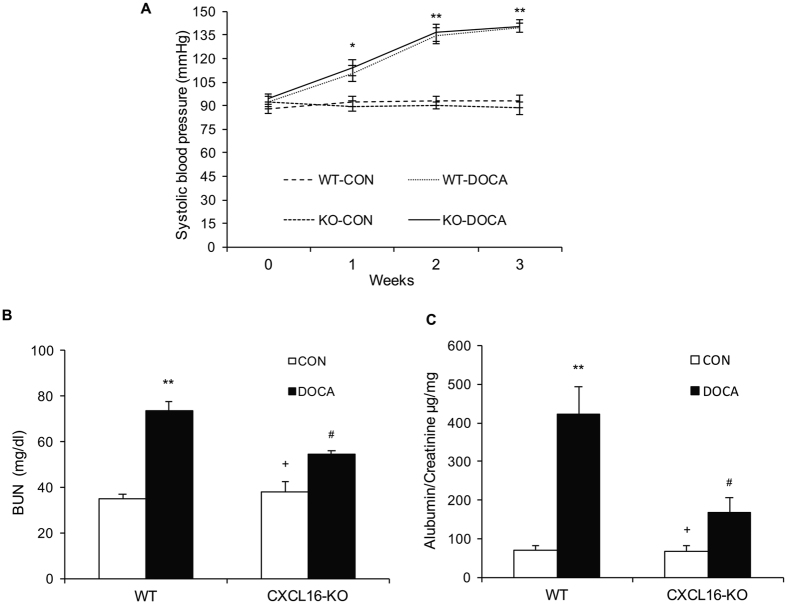
Effect of CXCL16 deficiency on blood pressure, renal function, and proteinuria. (**A**) Systolic blood pressure increased comparably between WT and CXCL16 KO mice following DOCA-salt treatment. *P < 0.05 and **P < 0.01 between DOCA-salt groups and vehicle control groups. (**B**) CXCL16 KO mice exhibited lower serum urea nitrogen compared with WT mice following DOCA-salt treatment. **P < 0.01 vs. WT-CON, ^+^*P* < 0.05 vs. KO-DOCA, ^#^*P* < 0.05 vs. WT-DOCA. (**C**) CXCL16 deficiency attenuated DOCA-salt-induced albuminuria. **P < 0.01 vs WT-CON, ^+^*P* < 0.05 vs KO-DOCA, ^#^*P* < 0.05 vs WT-DOCA. n = 6 in each group. BUN, blood urea nitrogen.

To examine the functional significance of CXCL16 induction in the kidney, WT and CXCL16 KO mice were subjected to uninephrectomy and treated with vehicle or DOCA-salt for 3 weeks. There were no significant differences in systolic blood pressure among the 4 groups at baseline. Systolic blood pressure in both WT and CXCL16 KO mice increased markedly following DOCA-salt treatment, which was comparable between the two DOCA-salt groups (Fig. 2A).

### Renal Function and Albuminuria

The blood urea nitrogen was elevated significantly in WT mice after treatment with DOCA-salt. In contrast, the blood urea nitrogen was significantly reduced in CXCL16 KO mice treated with DOCA-salt, indicating CXCL16 deficiency preserves renal function (Fig. 2B). Moreover, WT mice developed significant albuminuria after DOCA-salt treatment; whereas CXCL16 KO mice produced markedly less albuminuria in response to DOCA-salt (Fig. 2C).

### CXCL16 Deficiency Ameliorates Renal Injury and Fibrosis

**Figure 3:**
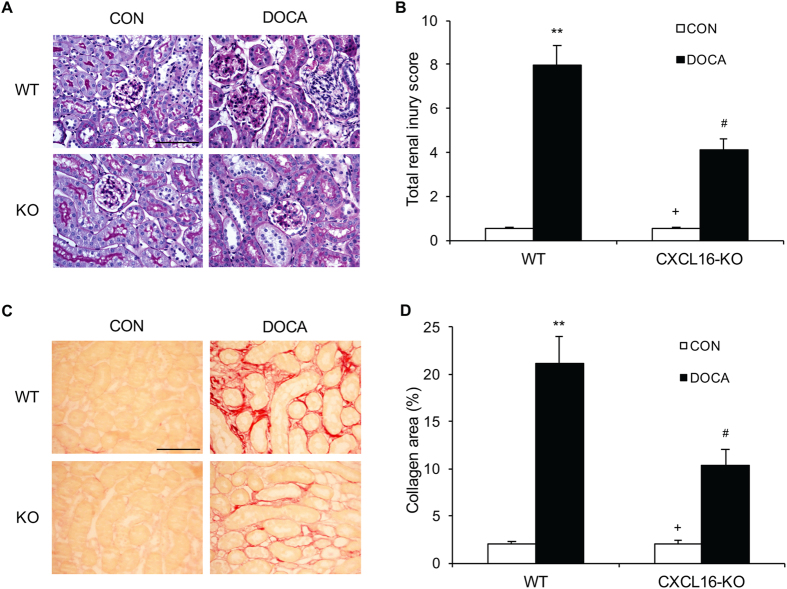
CXCL16 deficiency attenuates renal injury and fibrosis. (**A**) Representative photomicrographs of periodic acid-Schiff-stained kidney sections. Scale bar, 50 μm. (**B)** Quantitative assessment of renal injury in WT and CXCL16 KO mice. **P < 0.01 vs WT-CON, ^+^*P* < 0.05 vs KO-DOCA, ^#^*P* < 0.05 vs WT-DOCA. n = 6 in each group. (**C**) Representative photomicrographs of kidney sections stained with Sirius red for evaluation of total collagen deposition. Scale bar, 50 μm. (**D**) Quantitative analysis of interstitial collagen area in the kidneys. **P < 0.01 vs WT-CON, ^+^*P* < 0.05 vs KO-DOCA, ^#^*P* < 0.05 vs WT-DOCA. n = 6 in each group.

To investigate the role of CXCL16 in DOCA-salt–induced renal injury, PAS-stained kidney sections were examined for histological changes after 3 weeks of DOCA-salt treatment. There were minimal renal injuries in the 2 control groups (Fig. 3A,B). DOCA-salt treatment caused significant renal injury in the WT mice, which was significantly reduced in CXCL16 KO mice (Fig. 3A,B). Sirius red staining was performed to evaluate total collagen deposition in the kidney. WT mice treated with DOCA-salt exhibited a markedly collagen deposition in the kidney compared with WT controls (Fig. 3C,D). The degree of collagen deposition was substantially reduced in CXCL16 KO treated with DOCA-salt. These data suggest CXCL16 promotes the development of renal fibrosis in response to DOCA-salt hypertension (Fig. 3C,D).

### CXCL16 Deficiency Reduces ECM Protein Expression

**Figure 4:**
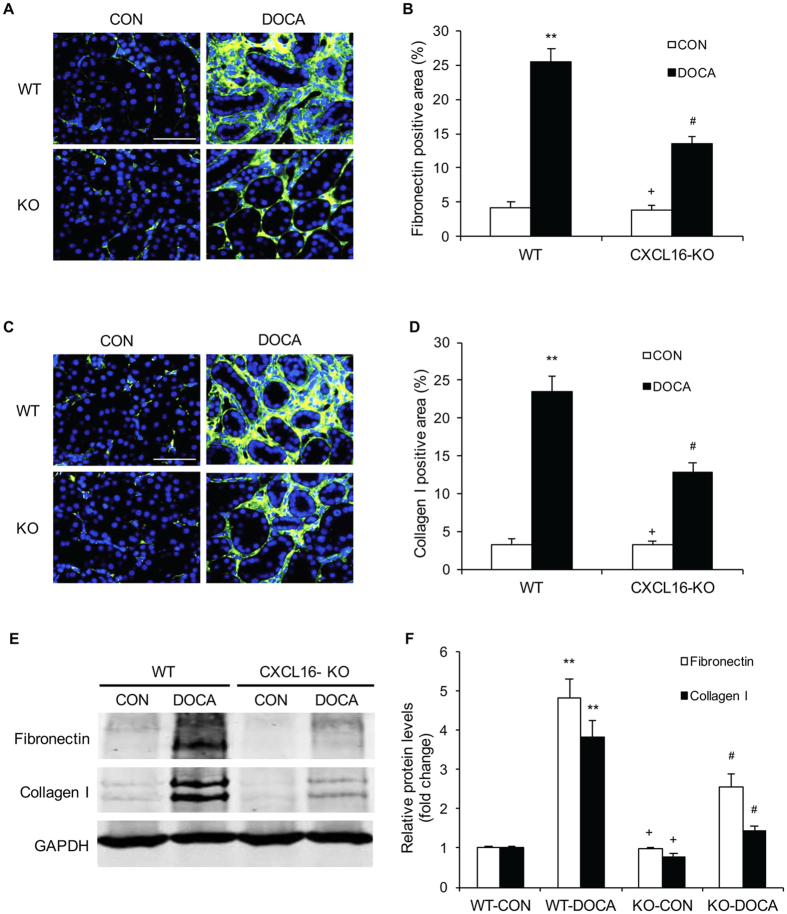
CXCL16 deficiency inhibits fibronectin and collagen I expression. (**A**) Representative photomicrographs of kidney sections stained for fibronectin. Scale bar, 50 μm. (**B**) Quantitative analysis of fibronectin-positive area in the kidneys. **P < 0.01 vs WT-CON, ^+^*P* < 0.05 vs KO-DOCA, ^#^*P* < 0.05 vs WT-DOCA. n = 6 in each group. (**C)** Representative photomicrographs of kidney sections stained for collagen I. Scale bar, 50 μm. (**D**) Quantitative analysis of collagen I-positive area in the kidneys. **P < 0.01 vs WT-CON, ^+^*P* < 0.05 vs KO-DOCA, ^#^*P* < 0.05 vs WT-DOCA. n = 6 in each group. (**E**) Representative Western blots show the protein levels of fibronectin and collagen I in the kidneys. (**F**) Quantitative analysis of fibronectin and collagen I protein expression in the kidneys of WT and CXCL16 KO mice. **P < 0.01 vs WT-CON, ^+^*P* < 0.05 vs KO-DOCA, ^#^*P* < 0.05 vs WT-DOCA. n = 6 in each group. GAPDH, glyceraldehyde-3-phosphate dehydrogenase.

To assess the role of CXCL16 in the production of ECM proteins, immunofluorescence staining was performed to detect the expression levels of collagen I and fibronectin, two major ECM proteins. The expression levels of collagen I and fibronectin in the kidneys of CXCL16 KO mice were markedly reduced compared with WT mice in response to DOCA-salt hypertension (Fig. 4A–D). In agreement with immunofluorescence staining, Western blot analysis demonstrated that CXCL16 deficiency reduced the expression levels of collagen I and fibronectin in the kidneys compared with WT mice after DOCA-salt treatment (Fig. 4E,F). These data indicate that CXCL16 plays a critical role in regulating ECM protein expression in the kidney in DOCA-salt hypertension.

### CXCL16 Deficiency Attenuates Myeloid Fibroblasts Accumulation

**Figure 5:**
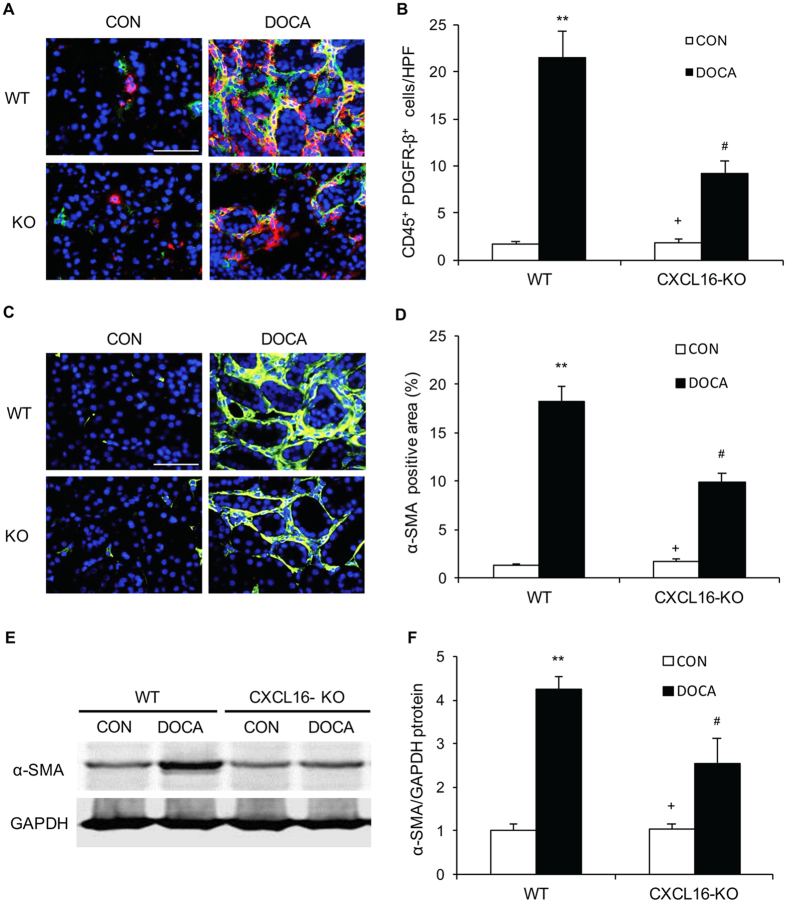
CXCL16 deficiency suppresses bone marrow–derived fibroblast accumulation and myofibroblast formation in the kidney. (**A)** Representative photomicrographs of kidney sections stained for CD45 (red), PDGFR-β (green), and DAPI (blue). Scale bar, 50 μm. (**B**) Quantitative analysis of CD45^+^ and PDGFR-β^+^ fibroblasts in kidneys. **P < 0.01 vs WT-CON, ^+^*P* < 0.05 vs KO-DOCA, ^#^*P* < 0.05 vs WT-DOCA. n = 6 in each group. (**C**) Representative photomicrographs of kidney sections stained for α-SMA. Scale bar, 50 μm. (**D**) Quantitative analysis of α-SMA positive area in kidneys. **P < 0.01 vs WT-CON, ^+^*P* < 0.05 vs KO-DOCA, ^#^*P* < 0.05 vs WT-DOCA. n = 6 in each group. (**E**) Representative Western blots show the levels of α-SMA protein expression in the kidneys. (**F**) Quantitative analysis of α-SMA protein expression in the kidneys. **P < 0.01 vs WT-CON,^+^*P* < 0.05 vs KO-DOCA, ^#^*P* < 0.05 vs WT-DOCA. n = 6 in each group. HPF, high-power field.

Recent studies have shown that myeloid fibroblasts contribute significantly to the development of renal fibrosis^10^,^12^,^17^,^18^,^19^,^20^,^21^,^22^,^23^,^24^, we then examined the role of CXCL16 in myeloid fibroblast accumulation. Kidney sections were stained for CD45, a hematopoietic marker, and PDGFR-β, a mesenchymal marker, and examined with a fluorescence microscope. The number of bone marrow–derived fibroblasts dual positive for CD45 and PDGFR-β was significantly reduced in the kidneys of CXCL16 KO mice treated with DOCA-salt compared with WT mice (Fig. 5A,B). These data indicate that CXCL16 plays an important role in recruiting bone marrow–derived fibroblasts into the kidney in response to DOCA-salt hypertension.

To determine if CXCL16 deficiency influences myofibroblast population, kidney sections were stained for α-smooth muscle actin (α-SMA), a marker of myofibroblasts, and examined with a fluorescence microscope. The results showed that the number of α-SMA positive myofibroblasts in the kidneys of CXCL16 KO mice treated with DOCA-salt was significantly decreased compared with WT mice (Fig. 5C,D). Consistent with these findings, Western blot analysis showed that the protein expression levels of α-SMA were significantly reduced in the kidneys of CXCL16 KO mice treated with DOCA-salt compared with WT mice (Fig. 5E,F). These results indicate that CXCL16 deficiency suppresses myofibroblast formation in the kidney.

### CXCL16 Deficiency Suppresses Macrophage and T Cell Infiltration

**Figure 6:**
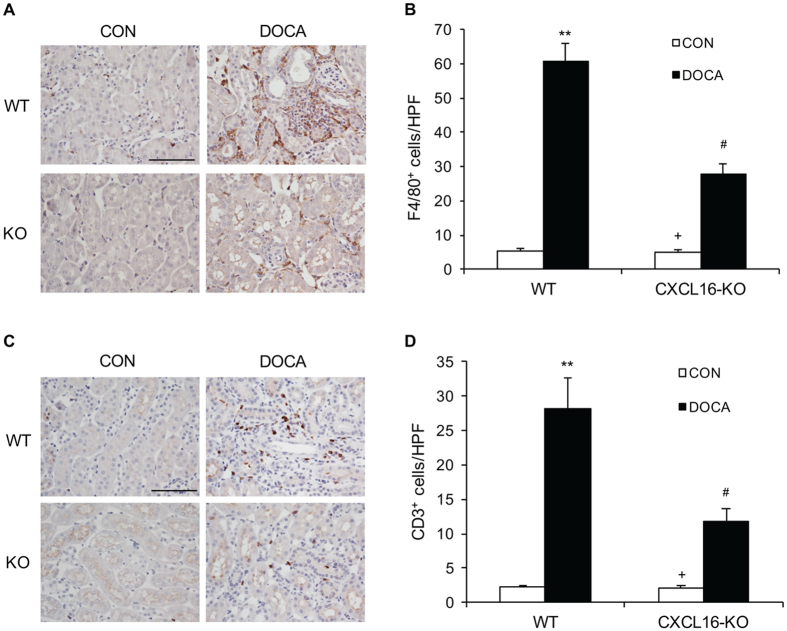
CXCL16 deficiency inhibits infiltration of macrophages and T cells in the kidney. (**A**) Representative photomicrographs of kidney sections stained for F4/80 (brown) and counterstained with hematoxylin (blue). Scale bar, 50 μm. (**B**) Quantitative analysis of F4/80^+^ macrophages in kidneys. **P < 0.01 vs WT-CON, ^+^*P* < 0.05 vs KO-DOCA, ^#^*P* < 0.05 vs WT-DOCA. n = 6 in each group. (**C**) Representative photomicrographs of kidney sections stained for CD3 (brown) and counterstained with hematoxylin (blue). Scale bar, 50 μm. (**D**) Quantitative analysis of CD3^+^ T cells in kidneys. **P < 0.01 vs WT-CON, ^+^*P* < 0.05 vs KO-DOCA, ^#^*P* < 0.05 vs WT-DOCA. n = 6 in each group. HPF, high power field.

To investigate if CXCL16 plays a role in inflammatory cell infiltration into the kidney, WT and CXCL16 KO mice were treated with DOCA-salt for 3 weeks. Kidney sections were stained for F4/80, a macrophage marker, and CD3, a T lymphocyte marker. The number of macrophages and T cells was markedly reduced in the kidneys of CXCL16 KO mice after DOCA-salt treatment compared with WT mice (Fig. 6). These data suggest that CXCL16 is involved in recruiting inflammatory cells into the kidney in DOCA-salt hypertension.

## Discussion

It is well established that patients with salt-sensitive hypertension are at a very high risk for the development of hypertensive renal injury and fibrosis^25^. Currently, there is no effective therapy and prevention for salt-induced hypertensive kidney damage^26^,^27^. Therefore, a better understanding the molecular mechanisms of salt-induced hypertensive renal injury is necessary. CXCL16 is a chemokine that plays an important role in regulating inflammation, tissue injury, and fibrosis^28^. However, its role in salt-sensitive hypertensive renal injury remains unknown. In the present study, we have demonstrated that: (1) CXCL16 is induced in the kidney in response to DOCA-salt hypertension; (2) genetic disruption of CXCL16 preserves renal function and protects against DOCA-salt-induced renal injury and fibrosis; (3) genetic disruption of CXCL16 inhibits myeloid fibroblast accumulation and myofibroblast formation in the kidney; (4) genetic disruption of CXCL16 suppresses inflammatory cell infiltration in the kidney. These results indicate that CXCL16 has a pivotal role in the pathogenesis of DOCA-salt-induced renal injury and fibrosis by regulating myeloid fibroblast accumulation and inflammation.

CXCL16 is a recently discovered chemokine that control leukocyte trafficking and can act as a scavenger receptor for oxidized low-density lipoprotein^28^. CXCL16 has a proinflammatory effect on renal proximal tubular cells and potentiates TWEAK-induced inflammatory responses^29^. We have recently shown that CXCL16 is upregulated in the kidney following obstructive injury and angiotensin II-induced hypertensive renal injury^10^,^21^. Genetic disruption of CXCL16 preserves renal function and reduces renal fibrosis^10^,^21^. In the present study, we have found that CXCL16 is induced in the kidney following DOCA-salt treatment. Therefore, we have investigated the role of CXCL16 in DOCA-salt-induced hypertensive renal injury using CXCL16 KO mice. Genetic disruption of CXCL16 has no effect on arterial blood pressure both at baseline and following DOCA-salt treatment. Importantly, genetic disruption of CXCL16 preserves kidney function, decreases urinary albumin excretion, and attenuates glomerular and vascular damage in the kidney after DOCA-salt administration. These data indicate that CXCL16 contributes to DOCA-salt-induced hypertensive renal injury.

Renal interstitial fibrosis is a hallmark of hypertensive kidney disease and the degree of interstitial fibrosis strongly correlates with rapidity of the progression of chronic kidney disease^30^. Renal interstitial fibrosis is manifested by substantial fibroblast activation and deposition of a large amount of ECM, which cause disruption of renal parenchyma and progressive loss of kidney function to end stage renal failure^4^. In the present study, we have shown that genetic disruption of CXCL16 significantly attenuates the development of renal interstitial fibrosis in DOCA-salt hypertension. Furthermore, the expression levels of fibronectin and collagen I, two major ECM proteins, were markedly reduced in CXCL16 KO mice with DOCA-salt hypertension. These data indicate that CXCL16 promotes renal fibrosis in response to DOCA-salt hypertension.

Activated fibroblasts are widely regarded as the principal matrix-producing cells that generate an excessive amount of ECM, including fibronectin and collagens^31^,^32^. The origin of activated fibroblasts has been intensively investigated. It is traditionally thought that activated fibroblasts arise from resident fibroblasts^33^,^34^. Recently, we and others have shown that activated fibroblasts may originate from bone marrow-derived fibroblast precursors^17^,^21^,^24^,^35^,^36^,^37^. Bone marrow–derived fibroblast precursors express hematopoietic markers such as CD45 and mesenchymal markers such as collagen and PDGFR-β^36^,^38^. We have demonstrated that bone marrow–derived fibroblasts migrate into the kidney following unilateral ureteral obstruction, ischemia-reperfusion injury, and angiotensin-induced renal injury and contribute significantly to the pathogenesis of renal fibrosis^10^,^12^,^21^,^23^,^36^,^37^,^38^. In the present study, we have shown that bone marrow–derived fibroblasts and myofibroblasts increase significantly in the kidney of WT mice in response to DOCA-salt hypertension. Genetic disruption of CXCL16 suppresses myeloid fibroblasts accumulation and myofibroblast formation and the development of renal interstitial fibrosis. These data suggest that CXCL16 plays an important role in the recruitment of bone marrow–derived fibroblasts into the kidney and the development of renal fibrosis in DOCA-salt hypertension.

Inflammatory and immune cells infiltration in the kidneys is regarded as a key event in hypertensive renal injury and fibrosis^11^,^39^,^40^,^41^,^42^,^43^. Chemokines interacting with their receptors are involved in recruiting inflammatory and immune cells into the kidney^12^,^38^,^44^. To determine the role of CXCL16 in recruitment of inflammatory and immune cells into the kidney in response to DOCA-salt hypertension, we have performed immunohistochemical staining for macrophages and T cells in the kidney. Our results have demonstrated that genetic disruption of CXCL16 significantly inhibits the infiltration of macrophages and T cells into the kidney in response to DOCA-salt hypertension. These data indicate that CXCL16 promotes renal injury through recruitment of macrophages and T cells into the kidney in DOCA-salt hypertension.

In summary, we have shown that CXCL16 plays an important role in the pathogenesis of renal injury and fibrosis in DOCA-salt hypertension. In response to DOCA-salt hypertension, CXCL16 recruits bone-marrow derived fibroblasts, macrophages, and T cells into the kidney, leading to renal injury and fibrosis. Our study suggests that CXCL16 signaling could serve as a novel therapeutic target for salt-sensitive hypertensive kidney disease.

## Material and Methods

### Animals

WT C57BL/6 mice were purchased from the Jackson Laboratory and CXCL16 KO mice on a C57BL/6 background were a generous gift from Dr. Shuhua Han at Baylor College of Medicine^10^,^21^. Mice were bred and maintained in the animal care facility of Baylor College of Medicine and had access to food and water ad libitum. All animal procedures were in accordance with national and international animal care and ethical guidelines and were approved by the Institutional Animal Care and Usage Committee at Baylor College of Medicine.

### DOCA-salt Hypertension

Male 8–10 weeks old mice were anesthetized by intraperitoneal injection of ketamine (80 mg/kg), xylazine (10 mg/kg), and acepromazine (3 mg/kg). After the left kidney of mice was removed, DOCA pellet (50 mg; Innovative Research of America) was implanted subcutaneously in the neck area. Mice receiving DOCA were given 1% sodium chloride to drink, and treatment with DOCA-salt continued for 3 weeks. Control mice underwent uninephrectomy without receiving DOCA pellet and saline.

### Blood pressure and heart rate

Systolic blood pressure (SBP) were measured in conscious mice using a tail cuff blood pressure system (Visitech Systems) as reported^10^,^12^,^45^.

### Albuminuria

Mice were placed into metabolic cages for urine collection at the end of experiments. Albumin and creatinine in the urine were measured using commercially available kits (EXOCELL, Inc)^10^,^12^.

### Renal Function

Blood urea nitrogen was detected fluorometrically as described^10^,^12^,^46^.

### Histopathologic Analysis

At the end of experiments, mice were perfused with phosphate-buffered saline to remove the blood. A portion of kidney tissue was fixed in 10% buffered formalin, embedded in paraffin, and cut at 4-μm thickness. After deparaffinization and rehydration, sections were stained with periodic acid-Schiff (PAS) and sirius red. The pathological abnormalities in the kidney were graded as described^47^, where 0 represented no abnormality and where 1, 2, 3, and 4 represented mild, moderate, moderately severe, and severe abnormalities, respectively. The sirius red-stained sections were scanned using a microscope equipped with a digital camera (Nikon Instruments Inc., Melville, NY), and quantitative evaluation was performed using NIS-Elements Br 3.0 software as described^10^,^12^,^21^. The sirius red-stained area was calculated as a percentage of the total area.

### Immunohistochemistry

Immunohistochemical staining was performed on paraffin sections. Antigen retrieval was performed with antigen unmasking solution (Vector Laboratories, Burlingame, CA) or proteinase K. Endogenous peroxidase activity was quenched with 3% H_2_O_2_ for 10 min. After blocking with 5% normal serum, sections were incubated with primary antibodies in a humidified chamber overnight. After washing, sections were incubated with appropriate secondary antibodies and ABC solution sequentially according to the instruction (Vector Laboratories). Immunoreactivity was then visualized by incubating sections in diaminobenzidine solution for an appropriate period of time. Nuclear staining was performed with hematoxylin. The slides were dehydrated, cleared, and mounted. The images from these slides were obtained and analyzed by NIS Element software (Nikon Instruments, Melville, NY) with Nikon microscope image system (Nikon Instruments)^10^,^12^.

### Immunofluorescence

Paraffin sections were stained with antibodies against fibronectin, collagen I, and α-SMA. After fixation and antigen retrieval, nonspecific binding was blocked with protein block (Dako, Carpinteria, CA). Kidney sections were then incubated with rabbit anti-collagen I antibody (Rockland Immunochemicals, Gilbertsville, PA), rabbit anti-fibronectin antibody (Sigma-Aldrich, St. Louis, MO), or rabbit anti-α-SMA antibody (Abcam, Cambridge, MA) followed by Alexa-488 conjugated donkey anti-rabbit antibody (Invitrogen, Carlsbad, CA). For double immunofluorescence staining, renal tissues were embedded in OCT compound, snap-frozen on dry ice, cut at 5 μm thickness, and mounted. Kidney sections were fixed and stained with rat anti-CD45 (BD Biosciences) and platelet-derived growth factor receptor (PDGFR)-β (Santa Cruz Biotechnology) followed by appropriate secondary antibodies sequentially. Slides were mounted with medium containing DAPI. Fluorescence intensity was visualized using a microscope equipped with a digital camera (Nikon Instruments Inc., Melville, NY). Quantitative evaluation of sections stained with antibodies to α-SMA, collagen I and fibronectin was performed using NIS-Elements Br 3.0 software. The fluorescence positive area was calculated as a percentage of the total area as described^10^,^12^,^21^.

### Western Blot Analysis

Protein was extracted using RIPA buffer containing cocktail proteinase inhibitors and quantified with a Bio-Rad protein assay. An equal amount of protein was separated on SDS-polycrylamide gels in Tris/SDS buffer system, and then transferred onto nitrocellulose membranes. The membranes were incubated with primary antibodies (CXCL16, collagen I, fibronectin and α-SMA) overnight, followed by incubation with appropriate fluorescence-conjugated secondary antibodies. The proteins of interest were analyzed using an Odyssey (LI-COR Bioscience, Lincoln, NE) IR scanner, and signal intensities were quantified using NIH Image/J software (National Institutes of Health, Bethesda, MD)^10^,^12^,^21^.

### Statistical Analysis

Data were expressed as mean ± SEM. Multiple group comparisons were performed by ANOVA followed by the Bonferroni procedure for comparison of means. Comparisons between two groups were analyzed by the two-tailed *t* test. A *P* value < 0.05 was considered statistically significant.

## Additional Information

**How to cite this article**: Liang, H. *et al*. CXCL16 Deficiency Attenuates Renal Injury and Fibrosis in Salt-Sensitive Hypertension. *Sci. Rep.* **6**, 28715; doi: 10.1038/srep28715 (2016).
